# Supplementary material for: Continuous Microfluidic Antisolvent Crystallization as a Bottom-Up Solution for the Development of Long-Acting Injectable Formulations
Source: Pharmaceutics. 2024 Mar 8;16(3):376. doi: 10.3390/pharmaceutics16030376 (PMC10974827; doi:10.3390/pharmaceutics16030376)
Supplement: Supplementary file 1 [file pharmaceutics-16-00376-s001.zip › pharmaceutics-2889408-supplementary.pdf]

Supplementary material

<u>**The following are the Supplementary data to this article:**</u>

Continuous Microfluidic Antisolvent Crystallization as a Bottom-Up Solution for the Development of Long-Acting Injectable Formulations

Snehashis Nandi [1,2,3], Laura Verstrepen [1,4], Mariana Hugo Silva [1,2,3], Luis Padrela [2,3], Lidia Tajber [2,5] and Alain Collas [1,*]

1 Janssen Pharmaceutica NV, Johnson & Johnson Innovative Medicine, 2340 Beerse, Belgium; snehashis.nandi@ul.ie (S.N.); lverstr2@its.jnj.com (L.V.); mhbbsilva@gmail.com (M.H.S.)
2 SSPC—The Science Foundation Ireland Research Centre for Pharmaceuticals, V94 T9PX, Limerick, Ireland;
luis.padrela@ul.ie (L.P.); ltajber@tcd.ie (L.T.)
3 Department of Chemical Sciences, Bernal Institute, University of Limerick, V94 T9PX Limerick, Ireland
4 Faculty of Pharmaceutical Sciences, University of Antwerp, 2000 Antwerp, Belgium
5 School of Pharmacy and Pharmaceutical Sciences, Trinity College Dublin, College Green, D02 PN40, Dublin, Ireland
* Correspondence: acollas@its.jnj.com

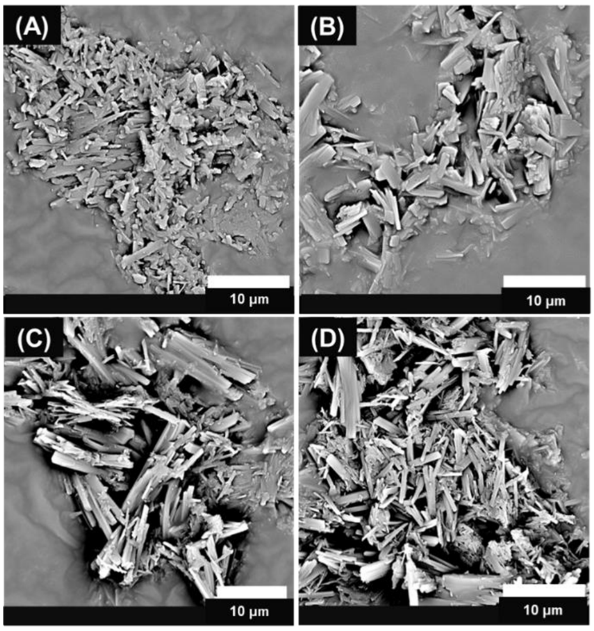

Figure S1: Scanning electron microscopy images of ITZ particles in suspension produced by Secoya microfluidic crystallization technology based continuous liquid antisolvent crystallization (SCT-CLASC) process with different S-AS ratios after 48 h of aging in an agitated crystallizer at 25°C. Using a Co-flow mixer with S:AS ratios of (A) 1:4, (B) 1:8, and using T-cross mixer with S:AS ratios of (C) 1:4, and (D) 1:8 respectively.

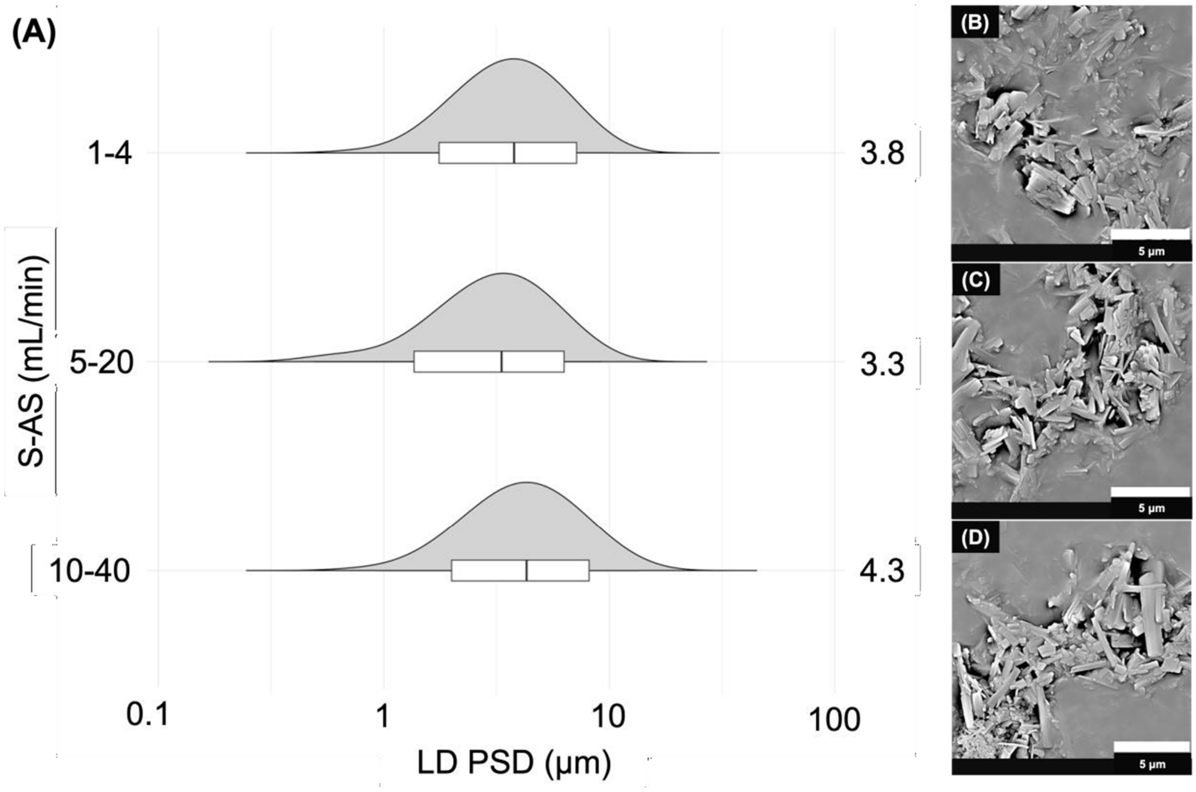

Figure S2: Effect of S-AS volumetric stream flow rate on the particle size distribution (PSD) of itraconazole (ITZ) in the generated suspensions (after 48 h of storage in an agitated crystallizer at 25°C) via Secoya microfluidic crystallization technology based continuous liquid antisolvent crystallization (SCT-CLASC) process using a Co-flow mixer, measured by laser diffraction (LD). The violin plots oriented with horizontal density curves depict the whole PSD. The results are presented in the overlaid box plot where the median corresponds to the D50 value mentioned on the right, the lower hinge to the D10, the higher hinge to the D90, and the whiskers span from D1 to D99. Scanning electron microscopy images of ITZ particles in suspension produced by SCT-CLASC process using a Co-flow mixer at constant S:AS ratio after 48 h of aging in an agitated crystallizer at 25°C, using S-AS volumetric stream flow rate of (B) 1-4 mL/min, (C) 5-20 mL/min, and (D) 10-40 mL/min, respectively.

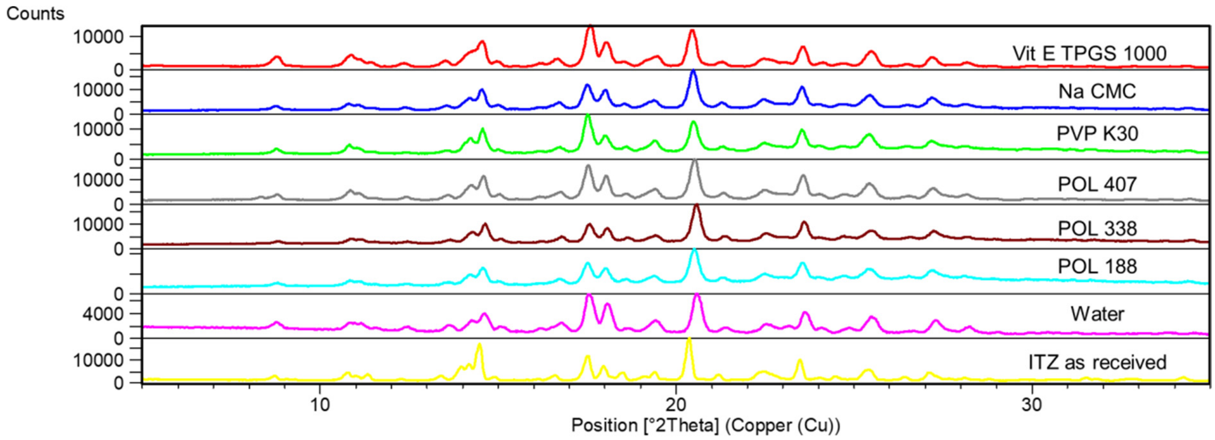

Figure S3: Powder X-ray diffraction spectra of itraconazole in suspension, generated via Secoya microfluidic crystallization technology based continuous liquid antisolvent crystallization (SCT-CLASC) process using six water-soluble stabilizers in water as antisolvent compared against reference water, after 48 h of storage in an agitated crystallizer at 25°C.

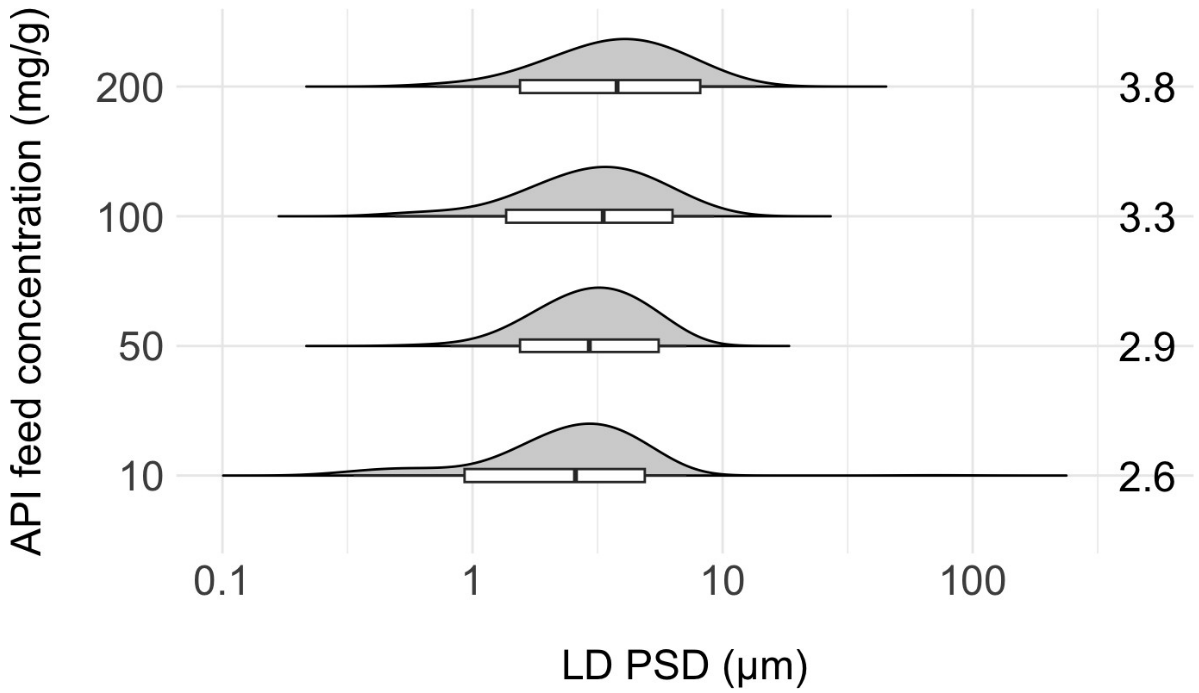

Figure S4: Role of starting API feed concentration on ITZ particle size distribution (PSD) in the generated suspensions (after 48 h of storage in an agitated crystallizer at 25°C) via Secoya microfluidic crystallization technology based continuous liquid antisolvent crystallization (SCT-CLASC) process using a Co-flow mixer, measured by laser diffraction (LD). The violin plots oriented with horizontal density curves depict the whole PSD. The results are presented in the overlaid box plot where the median corresponds to the D50 value mentioned on the right, the lower hinge to the D10, the higher hinge to the D90, and the whiskers span from D1 to D99.

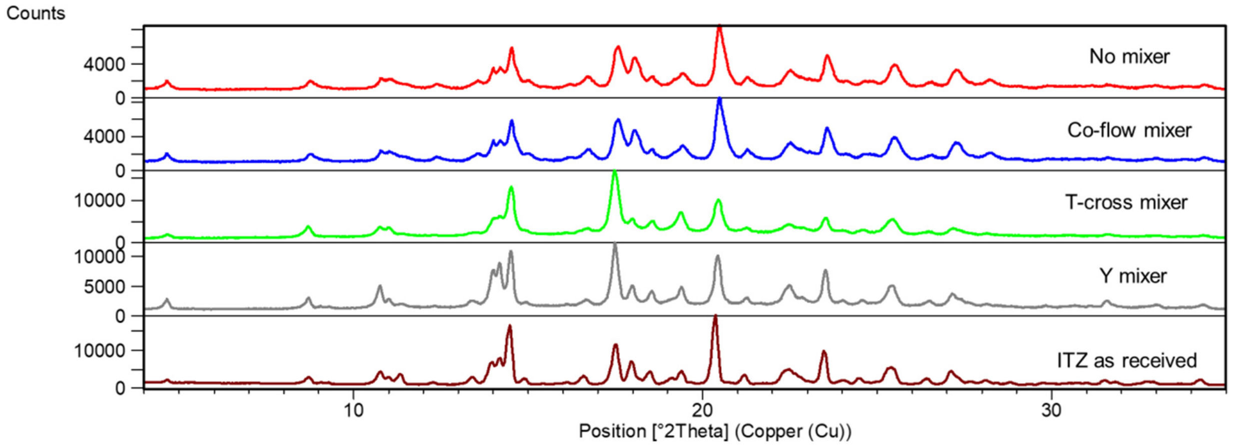

Figure S5: Powder X-ray diffraction spectra of itraconazole in suspension (solid loading of 20 mg/g) generated via Secoya microfluidic crystallization technology based continuous liquid antisolvent crystallization (SCT-CLASC) process using (A) No mixer (B) Co-flow mixer, (C) T-cross mixer, and (D) Y mixer, respectively after 48 h of storage in an agitated crystallizer at 25°C.

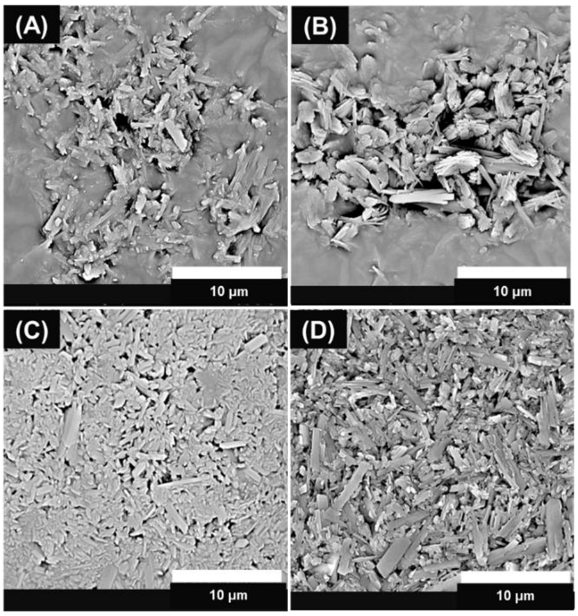

Figure S6: Scanning electron microscopy images of ITZ particles in suspension (solid loading of 20 mg/g) generated via Secoya microfluidic crystallization technology based continuous liquid antisolvent crystallization (SCT-CLASC) process using a Co-flow mixer (A) 0.5 % w/w Vit E TPGS 1000 solution as antisolvent, S-AS flow rate of 5 - 20 mL/min (B) 0.075 % w/w Vitamin E TPGS solution as antisolvent, S-AS flow rate of 5 - 20 mL/min, (C) 0.5 % w/w Vit E TPGS 1000 solution as antisolvent, S-AS flow rate of 10 - 40 mL/min, and (D) 0.075 % w/w Vit E TPGS 1000 solution as antisolvent, S-AS flow rate of 10 - 40 mL/min, respectively, measured after 48 h of storage in an agitated crystallizer at 25°C.

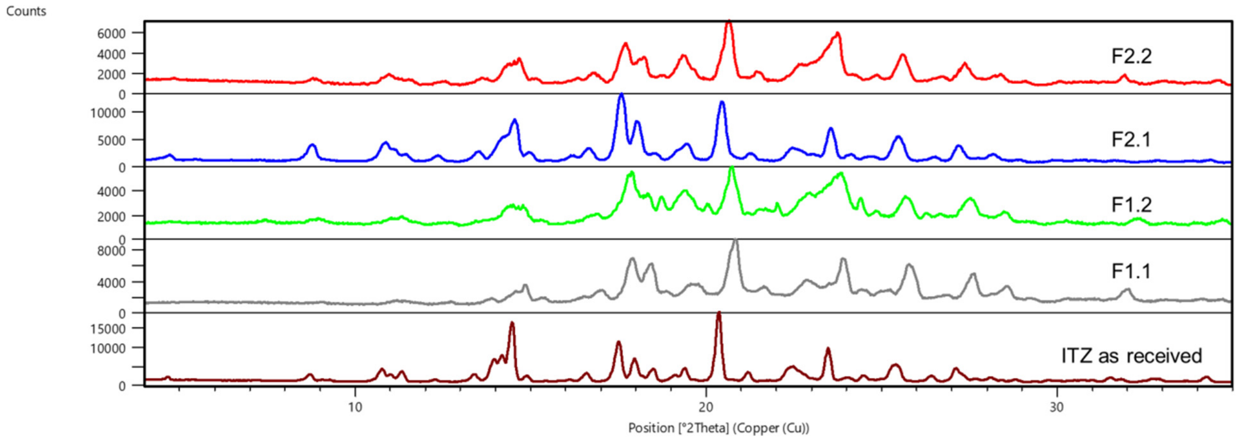

Figure S7: Powder X-ray diffraction spectra of four optimized itraconazole (ITZ) in suspension generated via Secoya microfluidic crystallization technology based continuous liquid antisolvent crystallization (SCT-CLASC) process after 48 h of storage in an agitated crystallizer at 25°C. F1.1: post-precipitation feed suspension solid loading of 40 mg/g, Vit E TPGS 1000 0.5 % w/w, F1.2: post-precipitation feed suspension solid loading of 20 mg/g, Vit E TPGS 1000 0.075 % w/w, F2.1: post-precipitation feed suspension solid loading of 40 mg/g, Vit E TPGS 1000 0.5 % w/w, F2.2: post-precipitation feed suspension solid loading of 40 mg/g, Vit E TPGS 1000 0.075 % w/w, and ITZ as received.

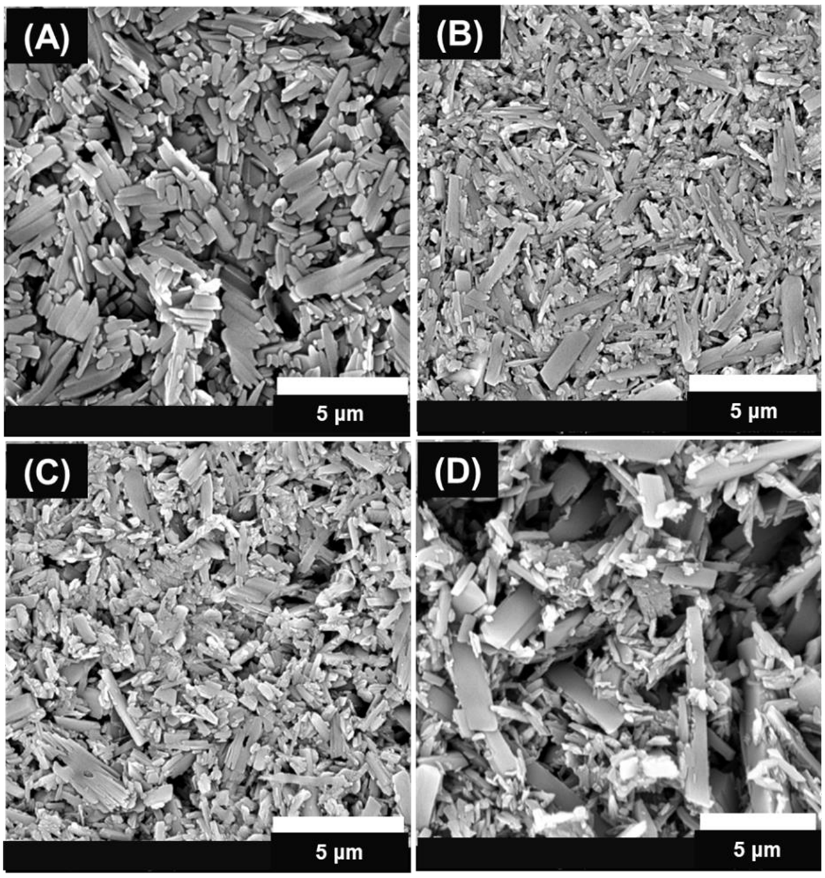

Figure S8: Scanning electron microscopy images of four optimized itraconazole in suspension generated via Secoya microfluidic crystallization technology based continuous liquid antisolvent crystallization (SCT-CLASC) process after 48 h of storage in an agitated crystallizer at 25°C. (A) F1.1: post-precipitation feed suspension solid loading of 40 mg/g, Vit E TPGS 1000 0.5 % w/w, (B) F1.2: post-precipitation feed suspension solid loading of 20 mg/g, Vit E TPGS 1000 0.075 % w/w, (C) F2.1: post-precipitation feed suspension solid loading of 40 mg/g, Vit E TPGS 1000 0.5 % w/w, (D) F2.2: post-precipitation feed suspension solid loading of 40 mg/g, Vit E TPGS 1000 0.075 % w/w.

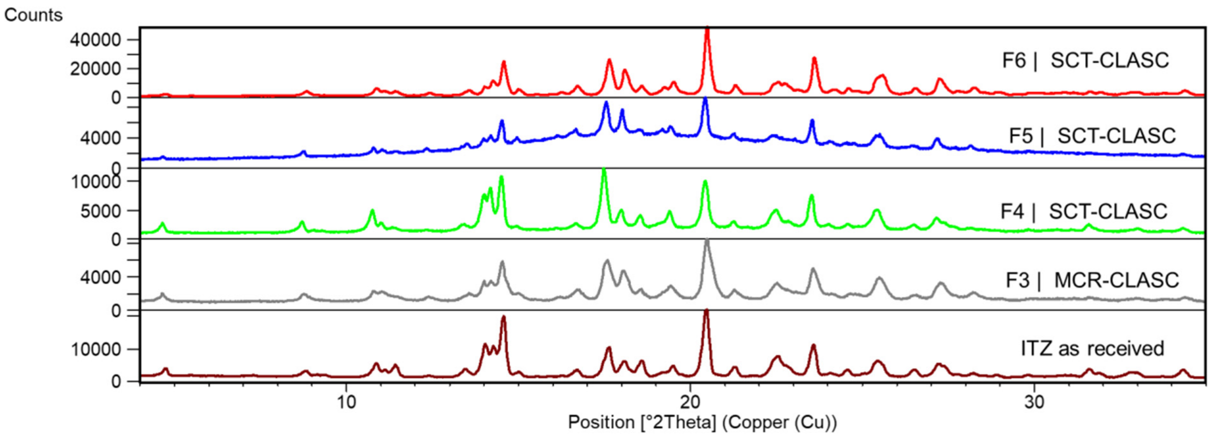

Figure S9: Overlay of powder Xray diffraction spectra of four optimized LAI suspensions of itraconazole (ITZ) (of final solid concentration 300 mg/g) in terms of particle size distribution (PSD) generated via downstream processing after MCR-CLASC and SCT-CLASC process. F6 / SCT-CLASC: post-precipitation feed suspension solid loading of 40 mg/g, Vit E TPGS 1000 0.075 % w/w, F5 / SCT-CLASC: post-precipitation feed suspension solid loading of 20 mg/g, Vit E TPGS 1000 0.075 % w/w, F4 / SCT-CLASC: post-precipitation feed suspension solid loading of 40 mg/g, Vit E TPGS 1000 0.5 % w/w, F3 / MCR-CLASC: post-precipitation feed suspension solid loading of 10 mg/g, Vit E TPGS 1000 0.5 % w/w, and ITZ as received.

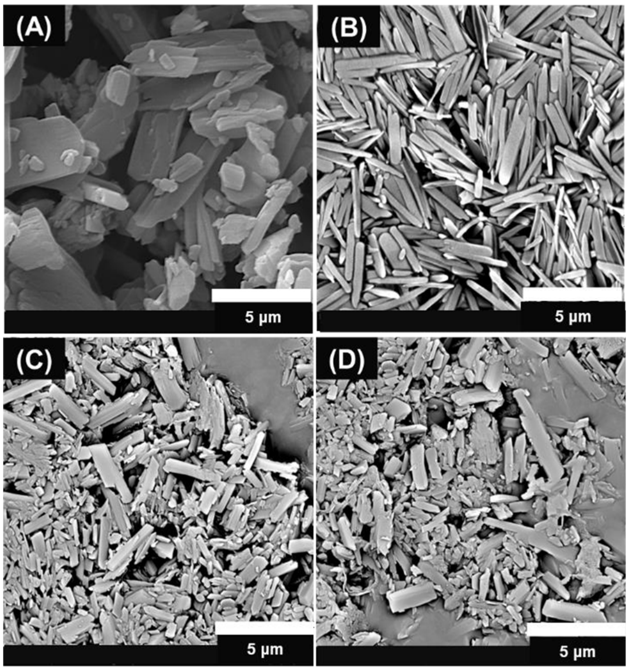

Figure S10: Scanning electron microscopy images of four optimized LAI suspensions of itraconazole (of final solid concentration 300 mg/mL) in terms of particle size distribution (PSD) generated via downstream processing after MCR-CLASC and SCT-CLASC process. (A) F3 / MCR-CLASC: post-precipitation feed suspension solid loading of 10 mg/g, Vit E TPGS 1000 0.5 % w/w, (B) F4 / SCT-CLASC: post-precipitation feed suspension solid loading of 40 mg/g, Vit E TPGS 1000 0.5 % w/w, (C) F5 / SCT-CLASC: post-precipitation feed suspension solid loading of 20 mg/g, Vit E TPGS 1000 0.075 % w/w, and (D) F6 / SCT-CLASC: post-precipitation feed suspension solid loading of 40 mg/g, Vit E TPGS 1000 0.075 % w/w.

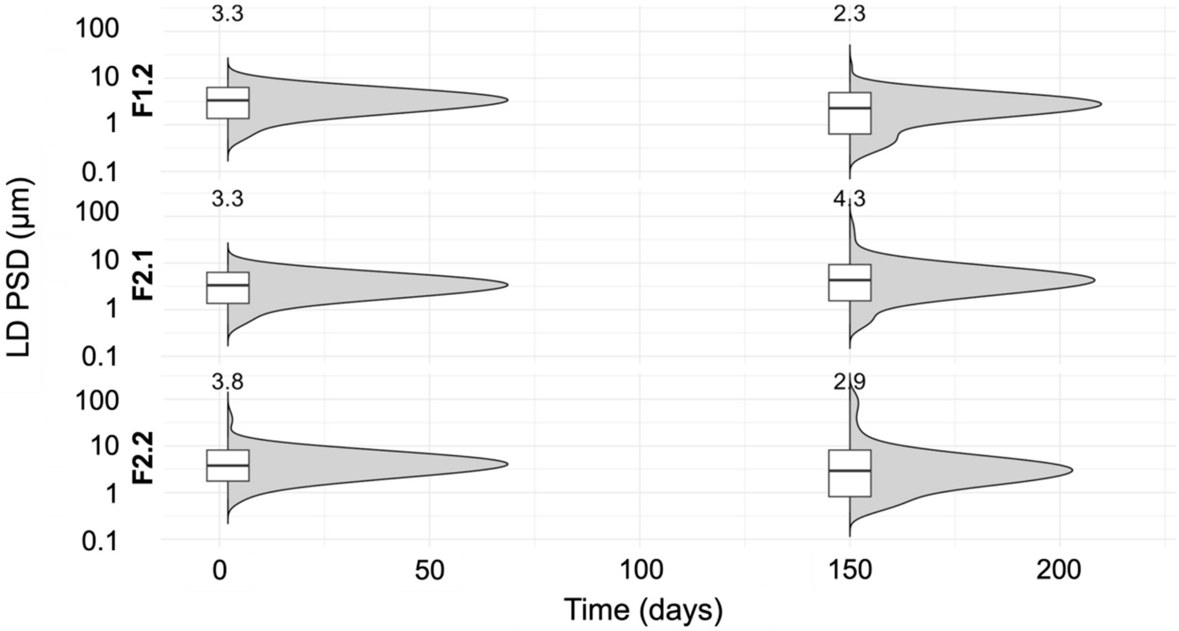

Figure S11: Particle size distribution (PSD) of ITZ in post-precipitation suspension after Secoya microfluidic crystallization technology based continuous liquid antisolvent crystallization (SCT-CLASC) process, measured by laser diffraction (LD) at regular time intervals (after 7, and 150 days) on stability stored at 25 °C. The violin plots oriented with vertical density curves depict the whole PSD. The results are presented in the overlaid box plot where the median corresponds to the D50, mentioned on the top of each violin plot, the lower hinge to the D10, the higher hinge to the D90, and the whiskers span from D1 to D99. ***F1.2****: post-precipitation feed suspension solid loading of 20 mg/g, Vit E TPGS 1000 0.075 % w/w,* ***F2.1****: post-precipitation feed suspension solid loading of 40 mg/g, Vit E TPGS 1000 0.5 % w/w,* ***F2.2****: post-precipitation feed suspension solid loading of 40 mg/g, Vit E TPGS 1000 0.075 % w/w.*

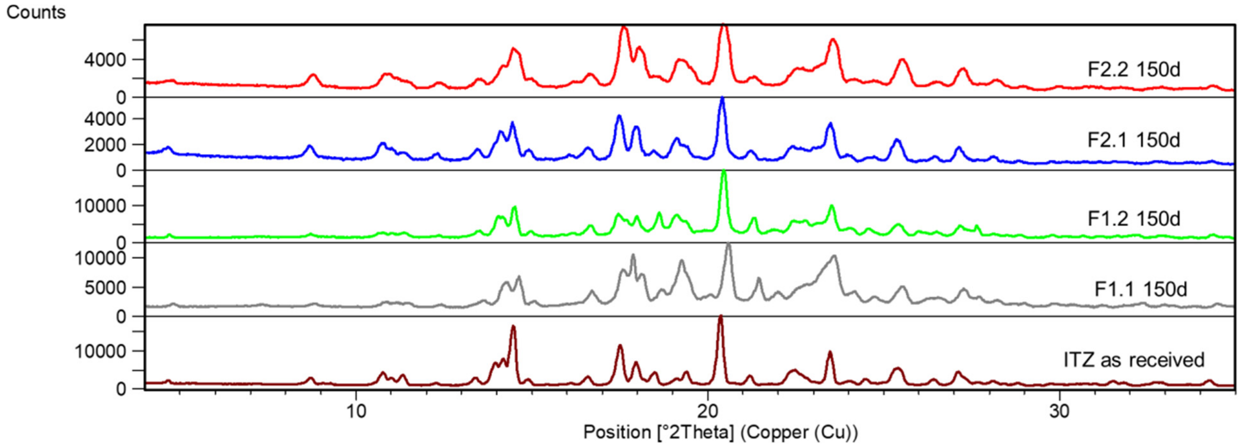

Figure S12: Powder X-ray diffraction spectra of three itraconazole (ITZ) in post-precipitation suspension after Secoya microfluidic crystallization technology based continuous liquid antisolvent crystallization (SCT-CLASC) process after 150 days (150d) on stability stored at 25 °C. ITZ as received, F1.1: post-precipitation feed suspension solid loading of 40 mg/g, Vit E TPGS 1000 0.5 % w/w, F1.2: post-precipitation feed suspension solid loading of 20 mg/g, Vit E TPGS 1000 0.075 % w/w, F2.1: post-precipitation feed suspension solid loading of 40 mg/g, Vit E TPGS 1000 0.5 % w/w, and F2.2: post-precipitation feed suspension solid loading of 40 mg/g, Vit E TPGS 1000 0.075 % w/w.

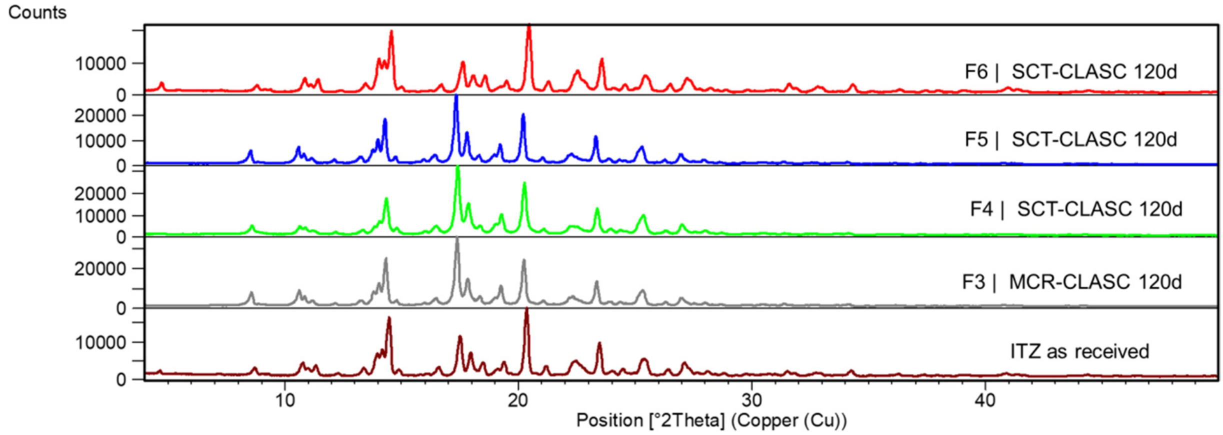

Figure S13: Powder X-ray diffraction spectra of three itraconazole (ITZ) in suspension via downstream processing after MCR-CLASC and SCT-CLASC process after 120 days (120d) on stability stored at 25 °C. ITZ as received, F3 / MCR-CLASC: post-precipitation feed suspension solid loading of 10 mg/g, Vit E TPGS 1000 0.5 % w/w, F4 / SCT-CLASC: post-precipitation feed suspension solid loading of 40 mg/g, Vit E TPGS 1000 0.5 % w/w, F5 / SCT-CLASC: post-precipitation feed suspension solid loading of 20 mg/g, Vit E TPGS 1000 0.075 % w/w, and F6 / SCT-CLASC: post-precipitation feed suspension solid loading of 40 mg/g, Vit E TPGS 1000 0.075 % w/w.
